# Ultrasound-promoted organocatalytic enamine–azide [3 + 2] cycloaddition reactions for the synthesis of ((arylselanyl)phenyl-1*H*-1,2,3-triazol-4-yl)ketones

**DOI:** 10.3762/bjoc.13.68

**Published:** 2017-04-11

**Authors:** Gabriel P Costa, Natália Seus, Juliano A Roehrs, Raquel G Jacob, Ricardo F Schumacher, Thiago Barcellos, Rafael Luque, Diego Alves

**Affiliations:** 1 Laboratório de Síntese Orgânica Limpa - LASOL - CCQFA - Universidade Federal de Pelotas - UFPel - P.O. Box 354 - 96010-900, Pelotas, RS, Brazil; 2 Laboratory of Biotechnology of Natural and Synthetic Products, Universidade de Caxias do Sul, Caxias do Sul, RS, Brazil; 3 Departamento de Quimica Organica, Universidad de Cordoba, Campus de Rabanales, Cordoba, Spain

**Keywords:** cycloadditions, organocatalysis, organoselenium compounds, sonochemistry, 1,2,3-triazoles

## Abstract

The use of sonochemistry is described in the organocatalytic enamine–azide [3 + 2] cycloaddition between 1,3-diketones and aryl azidophenyl selenides. These sonochemically promoted reactions were found to be amenable to a range of 1,3-diketones or aryl azidophenyl selenides, providing an efficient access to new ((arylselanyl)phenyl-1*H*-1,2,3-triazol-4-yl)ketones in good to excellent yields and short reaction times. In addition, this protocol was extended to β-keto esters, β-keto amides and α-cyano ketones. Selanyltriazoyl carboxylates, carboxamides and carbonitriles were synthesized in high yields at short times of reaction under very mild reaction conditions.

## Introduction

Substituted 1,2,3-triazoles are an interesting class of heterocyclic compounds distinguished by their biological activities [1–3] as well as in various fields of chemistry [4–15]. The most attractive way for their preparation is the thermal 1,3-dipolar cycloaddition of alkynes and azides, introduced by Huisgen which usually gives rise to a mixture of 1,4 and 1,5-isomers [16–19]. More recently, transition metal catalysts based on copper, ruthenium, silver and iridium salts have been used for this cycloaddition reaction [20–29].

Organocatalytic approaches based on β-enamine–azide or enolate–azide cycloadditions have been employed to synthesize 1,2,3-triazole scaffolds [30–32]. Depending on the organocatalyst employed, different carbonyl compounds could successfully generate an enamine or an enolate, and these species react as dipolarophiles with organic azides in organocatalyzed 1,3-dipolar cycloadditions. Our research group has demonstrated β-enamine–azide cycloaddition reactions for the synthesis of selenium-functionalized 1,2,3-triazoles [33–37]. Selanyltriazoyl carboxylates, carboxamides, carbonitriles or sulfones were synthesized in good to excellent yields using catalytic amounts of an organocatalyst.

**Figure 1:**
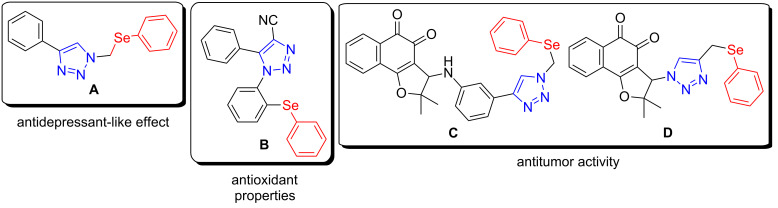
Biologically relevant selanyl-1,2,3-triazoles.

Organoselenium compounds are attractive synthetic targets because of their selective reactions [38–43], photophysical properties [44–49] and interesting biological activities [50–52]. An interesting class of molecules are the selanyl-1,2,3-triazoles [53–61] which can present some biological applications. As example, 4-phenyl-1-(phenylselanylmethyl)-1,2,3-triazole **A** (Se-TZ) demonstrated an antidepressant-like effect (Figure 1) [60]. In another example, 5-phenyl-1-(2-(phenylselanyl)phenyl)-1*H*-1,2,3-triazole-4-carbonitrile **B** (Se-TZCN) was reported to exhibit antioxidant activities in different in vitro assays (Figure 1) [36]. Selenanyl-quinone-based 1,2,3-triazoles **C** and **D** were synthesized and evaluated against six types of cancer cell lines. The synthesized compounds emerge as promising molecules for the therapeutic use of cancers overexpressing NQO1 (Figure 1) [61].

Thus, the search for efficient methods using appropriate and environmentally sound substrates for the preparation of selenium-functionalized 1,2,3-triazoles still remains a challenge in organic synthesis.

Ultrasonic irradiation has emerged in the past decades as a versatile tool in industrial and academic applications [62–67]. The use of sonication in organic synthesis (sonochemistry) is well documented and is generally considered as an environmentally sound energy source, comparatively less energy intensive to conventional heating and microwave irradiation, also able to reduce the number and quantities of side reaction products [62–67].

There are only a few contributions describing the use of sonochemistry for the preparation of functionalized 1,2,3-triazoles [68–74]. As a recent example, our research group described the use of sonochemistry in the organocatalytic enamine–azide [3 + 2] cycloadditions of β-oxo-amides with a range of substituted aryl azides providing and efficient access to new *N*-aryl-1,2,3-triazoyl carboxamides in good to excellent yields and short reaction times of [75].

**Scheme 1:**
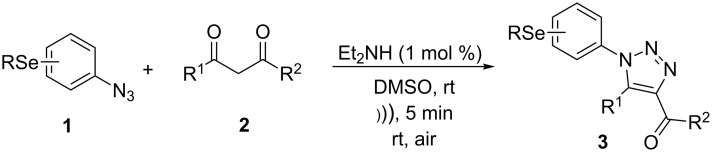
General scheme of the reaction.

However, to the best of our knowledge, the use of sonochemistry to synthesize complex selenium-functionalized 1,2,3-triazoles via organocatalytic enamine–azide cycloaddition has not been explored to date. As a continuation of our ongoing studies towards the development of new 1,2,3-triazoles bearing organoselenium moieties, this contribution was aimed to disclose a sonochemical approach for the organocatalyzed synthesis of ((arylselanyl)phenyl-1*H*-1,2,3-triazol-4-yl)ketones by reacting a range of 1,3-diketones with substituted aryl azidophenyl selenides (Scheme 1).

## Results and Discussion

**Scheme 2:**
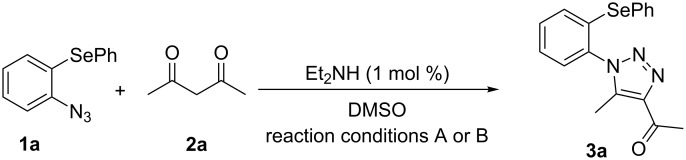
Comparative study of the conventional conditions and ultrasound irradiation. Conditions A: Reaction at 25 °C for 2 h (**3a**, 98%); Conditions B: Reaction under ultrasound irradiation (20% of the amplitude) at 25 °C for 20 min (**3a**, 92%).

Due to the fact that organocatalyzed β-enamine–azide cycloaddition reactions between azidophenyl aryl selenides and 1,3-diketones were not described, preliminary studies were attempted to react 2-azidophenyl phenyl selenide (**1a**) and 2,4-pentanedione (**2a**) as model reaction substrates. Based on our previous report on such reaction [33], a mixture of substrates **1a** (0.3 mmol) and **2a** (0.3 mmol) in DMSO (0.6 mL) was stirred at room temperature in the presence of 1 mol % of Et_2_NH as organocatalyst, providing an excellent yield (98%) of the desired product **3a** after 2 h (conditions A, Scheme 2).

With the aim to compare the effect of different energy sources in this β-enamine–azide cycloaddition, the reaction between substrates **1a** and **2a** in DMSO using Et_2_NH (1 mol %) was also performed under ultrasound irradiation.

The reaction performed under ultrasound irradiation with 20% of the amplitude for 20 minutes (followed by TLC until the total consumption of the starting materials) yielded product **3a** in 92% (conditions B, Scheme 2). Inspired by results described under conditions B, we performed additional experiments using ultrasound irradiation with Et_2_NH as organocatalyst (Table 1).

**Table 1:** Optimization of reaction conditions.^a^

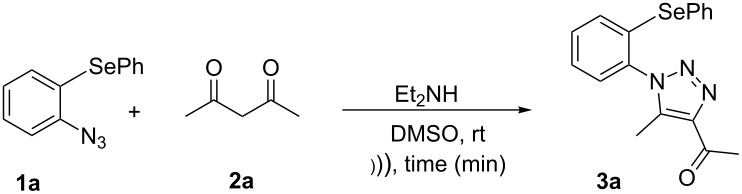				

Entry	Amplitude	Et_2_NH (mol %)	Time (min)	Yield **3a** (%)^b^

1	20	1	20	92
2	25	1	20	92
3	30	1	20	93
4	40	1	20	96
5	20	1	10	70
6	40	1	10	95
7	40	1	5	93
8	40	0.5	25	85
9	40	0.1	60	n.d.
10	40	–	60	n.d.
11^c^	40	1	5	27
12^d^	40	1	5	85

^a^Reactions were performed with 2-azidophenyl phenyl selenide (**1a**, 0.3 mmol) and 2,4-pentanedione (**2a**, 0.3 mmol) in DMSO (0.6 mL) as solvent under ultrasound irradiation at 25 °C. ^b^Yields are given for isolated products. ^c^Reaction was performed with L-proline as a catalyst. ^d^Reaction was performed with pyrrolidine (1 mol %). n.d.: not detected.

Initially, substrates **1a** and **2a** were reacted in DMSO under ultrasound irradiation for 20 min using different amplitudes (Table 1, entries 1–4). We observed that the desired product **3a** was obtained in excellent yields in all reactions. However, product yield of **3a** decreased to 70% (Table 1, entry 5) in 10 minutes under 20% sonochemistry amplitude. To our delight, reactions performed using 40% of amplitude during 10 or 5 min gave excellent yields of selanyltriazole **3a** (Table 1, entries 6 and 7). We observed that the amplitude effect could be correlated to the product formation time, since that in reaction carried out in 40% of amplitude the yield of compound **3a** was excellent (93%) after 5 min reaction time (Table 1, entry 5 vs 7). A slight decrease in reaction yields could be observed after decreasing the loading of organocatalyst to 0.5 mol % (Table 1, entry 8). Finally, in blank runs (in the absence of organocatalyst) or performed using 0.1 mol % of catalyst the reaction did not occur, even under sonication for 60 min using 40% of amplitude (Table 1, entries 9 and 10). Reactions performed with other catalysts (L-proline and pyrrolidine) gave lower yields of **3a** than those using 1 mol % of Et_2_NH (Table 1, entry 7 vs entries 11 and 12).

From Table 1, optimum reaction conditions to obtain 1-(5-methyl-1-(2-(phenylselanyl)phenyl)-1*H*-1,2,3-triazol-4-yl)ethan-1-one (**3a**) were clearly present in entry 7, in which a mixture of azidophenyl phenyl selenide (**1a**, 0.3 mmol), 2,4-pentanedione (**2a**, 0.3 mmol) and Et_2_NH (1 mol %) in DMSO (0.6 mL) was sonicated using 40% of amplitude at room temperature for 5 minutes. In order to extend the scope of the reaction, optimum reaction conditions were extended to other 1,3-diketones **2a–e** with different substitution patterns (Table 2). High yields of desired 1,2,3-triazoles were obtained using β-diketones **2a**, **2b** and **2c** bearing methyl, ethyl and phenyl substituents (Table 2, entries 1–3). However, we observed that the steric hindrance effect in 2,2,6,6-tetramethyl-3,5-heptanedione **2d** displays an important role in the overall reaction and only traces of product **3d** was observed (Table 2, entries 1–3 vs 4). Unfortunately, no reaction occurred when cyclic β-diketone **2e** was employed as substrate (Table 2, entry 5). We next evaluated the reactivity of 2,4-pentanedione (**2a**) with different functionalized aryl azidophenyl selenides **1b–f** under identical reaction conditions. Aryl azidophenyl selenides containing either an EDG or an EWG on the aromatic ring delivered the expected selanyltriazoles **3f–i** in good isolated yields (Table 2, entries 6–9). However, a decrease in yield was observed when the reaction was performed with aryl azidophenyl selenide containing a –CF_3_ group (Table 2, entry 9). In addition, 4-azidophenyl phenyl selenide (**1f**) was treated with 2,4-pentanedione (**2a**) to afford the desired product **3j** in 92% yield as a mixture of regioisomers (6:1) (Table 2, entry 10).

**Table 2:** Scope of substrates: Variation of the aryl azidophenyl selenides **1** and 1,3-diketones **2**.^a^

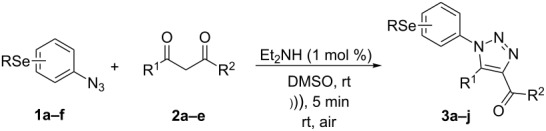				

Entry	Aryl azidophenyl selenides **1**	1,3-Diketone **2**	Product **3**	Isolated Yield (%)^b^

1	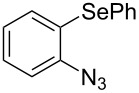 **1a**	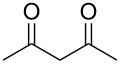 **2a**	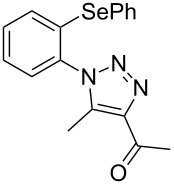 **3a**	93
2	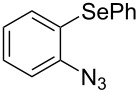 **1a**	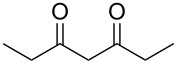 **2b**	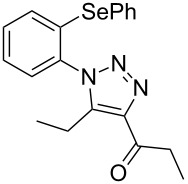 **3b**	91
3	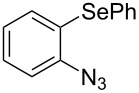 **1a**	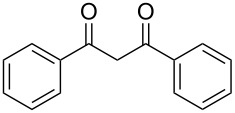 **2c**	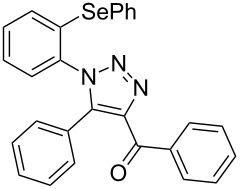 **3c**	85
4	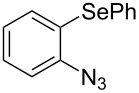 **1a**	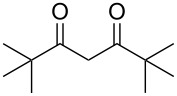 **2d**	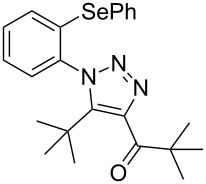 **3d**	traces
5	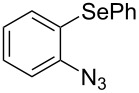 **1a**	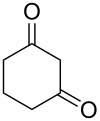 **2e**	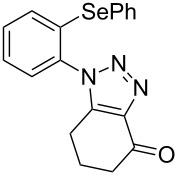 **3e**	n.d.
6	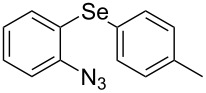 **1b**	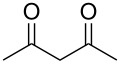 **2a**	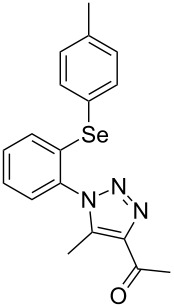 **3f**	74
7	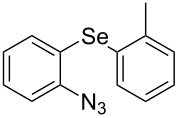 **1c**	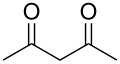 **2a**	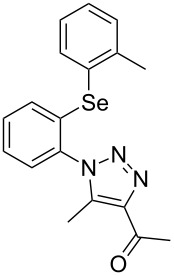 **3g**	84
8	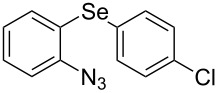 **1d**	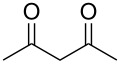 **2a**	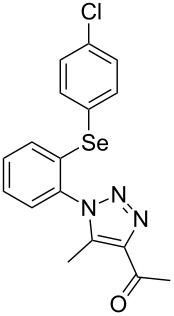 **3h**	87
9	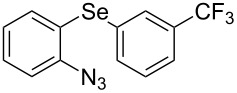 **1e**	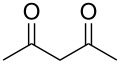 **2a**	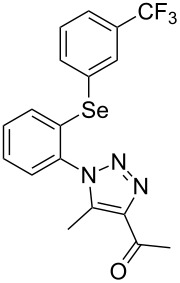 **3i**	56
10	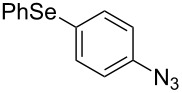 **1f**	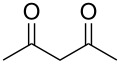 **2a**	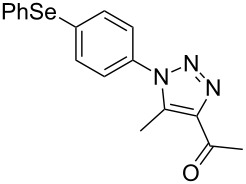 **3j**	92

^a^Reactions were performed with aryl azidophenyl selenides **1a–f** (0.3 mmol) and 1,3-diketones **2a–e** (0.3 mmol), using Et_2_NH (1 mol %) as catalyst in DMSO (0.6 mL) as solvent under ultrasound irradiation (40% of amplitude) at room temperature for 5 min. ^b^Yields are given for isolated products. n.d.: not detected.

**Scheme 3:**
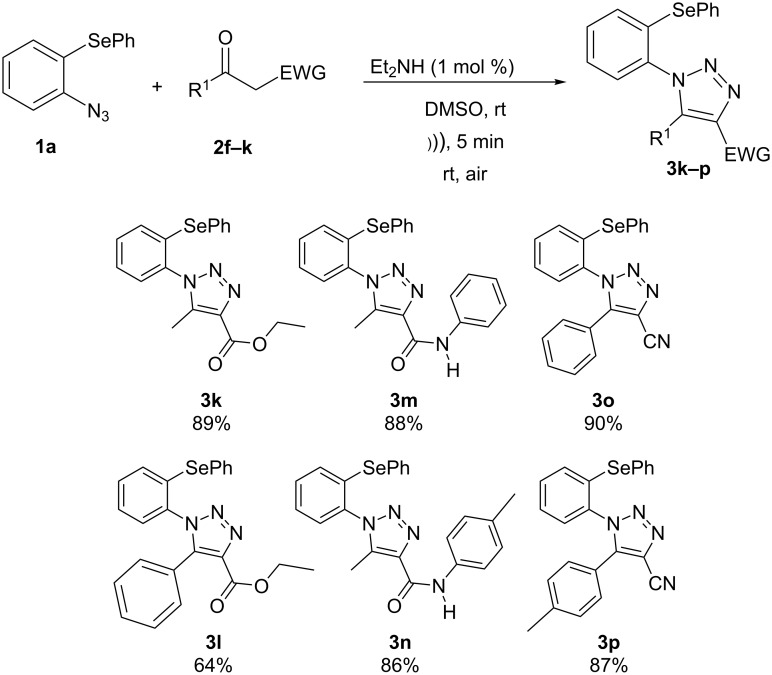
Reaction of 2-azidophenyl phenyl selenide **1a** with activated ketones **2f–k**.

In addition, the possibility to perform the reaction of 2-azidophenyl phenyl selenide (**1a**) with β-keto-esters, β-keto-amides and α-cyano-ketones **2f–k** was also investigated. The reaction conditions optimized for 1,3-diketone **2a** were employed, but independently using as substrates ethyl acetoacetate (**2f**), ethyl benzoylacetate (**2g**), 3-oxo-*N*-phenylbutanamide (**2h**), 3-oxo-*N*-(*p*-tolyl)butanamide (**2i**), benzoylacetonitrile (**2j**) and 4-toluoylacetonitrile (**2k**). The corresponding esters **3k**,**l** [33], amides **3m**,**n** [34] and nitriles **3o**,**p** [36] were obtained in good yields (Scheme 3) after 5 minutes reaction under ultrasound irradiation (40% of amplitude) at room temperature. Comparing these results with already published ones under conventional conditions, our methodology using ultrasound irradiation affords the products in 5 minutes and in comparable yields while the other methods mostly provide the products in times above 60 minutes [33–34,36].

## Conclusion

In summary, we have described the use of sonochemistry in the organocatalytic enamine–azide [3 + 2] cycloaddition between 1,3-diketones and aryl azidophenyl selenides. These sonochemical promoted reactions were found to be amenable to a range of 1,3-diketones or aryl azidophenyl selenides, providing an efficient access to novel selenium-containing 1,2,3-triazole compounds in good to excellent yields, in a few minutes of reaction at room temperature. The protocol was extended to activated ketones and selanyltriazoyl carboxylates, with carboxamides and carbonitriles synthesized in high yields and short times of reaction.

## Experimental

### General information

The reactions were monitored by TLC carried out on Merck silica gel (60 F_254_) by using UV light as visualizing agent and 5% vanillin in 10% H_2_SO_4_ and heat as developing agents. Baker silica gel (particle size 0.040–0.063 mm) was used for flash chromatography. A Cole Parmer-ultrasonic processor Model CPX 130, with a maximum power of 130 W, operating at an amplitude of 40% and a frequency of 20 kHz was used. The temperature of the reaction was monitored using an Incoterm digital infrared thermometer Model Infraterm (Brazil) (in most reactions the temperature was in the range between 60 and 65 °C). Proton nuclear magnetic resonance spectra (^1^H NMR) were obtained at 400 MHz on Bruker DPX 400 spectrometer. Spectra were recorded in CDCl_3_ solutions. Chemical shifts are reported in ppm, referenced to tetramethylsilane (TMS) as the external reference. Coupling constants (*J*) are reported in Hertz. Abbreviations to denote the multiplicity of a particular signal are s (singlet), d (doublet), t (triplet), q (quartet) and m (multiplet). Carbon-13 nuclear magnetic resonance spectra (^13^C NMR) were obtained at 100 MHz on Bruker DPX 400 spectrometer. Chemical shifts are reported in ppm, referenced to the solvent peak of CDCl_3_. Low-resolution mass spectra were obtained with a Shimadzu GC-MS-QP2010 mass spectrometer. High resolution mass spectra (HRMS) were recorded on a Bruker Micro TOF-QII spectrometer 10416.

### General procedure for the synthesis of selanyltriazoles **3a–r** under ultrasound irradiation

Aryl azidophenyl selenides **1a–f** (0.3 mmol), activated ketones **2a–k** (0.3 mmol), Et_2_NH (1 mol %) and DMSO (0.6 mL) were added to a glass tube. The ultrasound probe was placed in a glass vial containing the reaction mixture. The amplitude of the ultrasound waves was fixed in 40%. Then, the reaction mixture was sonicated for 5 min. The crude product obtained was subsequently purified by column chromatography on silica gel using a mixture of hexane/ethyl acetate (5:1) as eluent to afford the desired products **3a–p**.

## Supporting Information

File 1 Experimental and analytical data.
