# Supplementary material for: Ultrasound-promoted organocatalytic enamine–azide [3 + 2] cycloaddition reactions for the synthesis of ((arylselanyl)phenyl-1H-1,2,3-triazol-4-yl)ketones
Source: Beilstein J Org Chem. 2017 Apr 11;13:694–702. doi: 10.3762/bjoc.13.68 (PMC5405684; doi:10.3762/bjoc.13.68)
Supplement: File 1 — Experimental and analytical data. [file Beilstein_J_Org_Chem-13-694-s001.pdf]

Supporting Information

for

Ultrasound-promoted organocatalytic enamine–azide [3 + 2] cycloaddition reactions for the synthesis of ((arylselanyl)phenyl-1*H*-1,2,3-triazol-4-yl)ketones

Gabriel P. Costa[1], Natália Seus[1], Juliano A. Roehrs[1], Raquel G. Jacob[1], Ricardo F. Schumacher[1], Thiago Barcellos[2], Rafael Luque*[3] and Diego Alves*[1]

Address: [1]Laboratório de Síntese Orgânica Limpa - LASOL - CCQFA - Universidade Federal de Pelotas - UFPel - P.O. Box 354 - 96010-900, Pelotas, RS, Brazil, [2]Laboratory of Biotechnology of Natural and Synthetic Products, Universidade de Caxias do Sul, Caxias do Sul, RS, Brazil and [3]Departamento de Quimica Organica, Universidad de Cordoba, Campus de Rabanales, Cordoba, Spain.

Email: Diego Alves - diego.alves@ufpel.edu.br; Rafael Luque - q62alsor@uco.es

*Corresponding author

Experimental and analytical data

Contents

General information...S2

General procedure for the synthesis of selanyltriazoles 3a–p under ultrasound irradiation…………………………………………………………………………………..S2

Spectral data of the products..S3

Selected spectra……......................………..………………………………….........……....S7

General Information: The reactions were monitored by TLC carried out on Merck silica gel (60 F_{254}) by using UV light as visualizing agent and 5% vanillin in 10% H_2SO_4 and heat as developing agents. Baker silica gel (particle size 0.040–0.063 mm) was used for flash chromatography. A Cole Parmer-ultrasonic processor Model CPX 130, with a maxim power of 130 W, operating at an amplitude of 40% and a frequency of 20 kHz was used. The temperature of the reaction was monitored using an Incoterm digital infrared thermometer Model Infraterm (Brazil) (in most reactions the temperature was in the range between 60 and 65 °C). Proton nuclear magnetic resonance spectra (^1H NMR) were obtained at 400 MHz on Bruker DPX 400 spectrometer. Spectra were recorded in $CDCl_3$ solutions. Chemical shifts are reported in ppm, referenced to tetramethylsilane (TMS) as the external reference. Coupling constants (*J*) are reported in Hertz. Abbreviations to denote the multiplicity of a particular signal are s (singlet), d (doublet), t (triplet), q (quartet) and m (multiplet). Carbon-13 nuclear magnetic resonance spectra (^{13}C NMR) were obtained at 100 MHz on Bruker DPX 400 spectrometer. Chemical shifts are reported in ppm, referenced to the solvent peak of $CDCl_3$. Low-resolution mass spectra were obtained with a Shimadzu GC-MS-QP2010 mass spectrometer. High resolution mass spectra (HRMS) were recorded on a Bruker Micro TOF-QII spectrometer 10416.

General procedure for the synthesis of selanyl-triazoles 3a–r under ultrasound irradiation: Aryl azidophenyl selenides **1a–f** (0.3 mmol), activated ketones **2a–k** (0.3 mmol), Et_2NH (1 mol %) and DMSO (0.6 mL) were added to a glass tube. The ultrasound probe was placed in a glass vial containing the reaction mixture. The amplitude of the ultrasound waves was fixed in 40%. Then, the reaction mixture was sonicated for 5 min. The crude product obtained was subsequently purified by column chromatography on silica gel using a mixture of hexane/ethyl acetate (5:1) as eluent to afford the desired products **3a–p**. Spectral data for the products prepared are listed below. The data of obtained compounds **3k–p** are in agreement with the already published data [1,2,3].

[1] Seus, N.; Gonçalves, L. C. C.; Deobald, A. M.; Savegnago, L.; Alves, D.; Paixão, M. W. *Tetrahedron* **2012**, *68*, 10456.
[2] Seus, N.; Goldani, B.; Lenardão, E. J.; Savegnago, L.; Paixão, M. W.; Alves, D. *Eur. J. Org. Chem.* **2014**, 1059.

Spectral data of the products

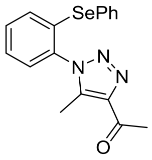

1-(5-Methyl-1-(2-(phenylselanyl)phenyl)-1*H*-1,2,3-triazol-4-yl)ethan-1-one (3a). Yield: 0.099 g (93%); yellow solid; mp 81-82 ºC. ^{1}H NMR ($CDCl_3$, 400 MHz) δ = 7.47-7.28 (m, 9H), 2.76 (s, 3H), 2.44 (s, 3H). ^{13}C NMR ($CDCl_3$, 100 MHz) δ = 194.09, 143.08, 138.47, 135.19, 135.08, 134.88, 133.15, 132.80, 131.17, 129.58, 128.72, 127.80, 127.70, 27.67, 9.61. MS *m/z* (relative intensity): 357 (40), 355 (20), 286 (20), 271 (19), 252 (98), 250 (51), 232 (33), 207 (100), 172 (25), 157 (17), 152 (68), 130 (33), 77 (58), 51 (30). HRMS calcd for $C_{17}H_{16}N_3OSe$ [M + H]$^+$: 358.0459. Found: 358.0467.

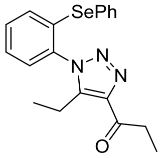

1-(5-Ethyl-1-(2-(phenylselanyl)phenyl)-1H-1,2,3-triazol-4-yl)propan-1-one (3b). Yield: 0.105 g (91%); yellow oil. ^{1}H NMR ($CDCl_3$, 400 MHz) δ = 7.52-7.49 (m, 2H), 7.40-7.28 (m, 7H), 3.28 (q, *J* = 7.3 Hz, 2H), 2.89 (q, *J* = 7.5 Hz, 2H), 1.27 (t, *J* = 7.3 Hz, 3H), 1.13 (t, *J* = 7.3 Hz, 3H). ^{13}C NMR ($CDCl_3$, 100 MHz) δ = 197.07, 143.77, 142.31, 135.49, 134.69, 133.58, 132.69, 131.29, 129.75, 128.93, 127.74, 127.71, 127.55, 33.27, 17.21, 12.77, 7.82. MS (relative intensity) m/z: 385 (52), 357 (8), 300 (16), 280 (71), 252 (16), 232 (42), 221 (48), 206 (27), 184 (11), 152 (64), 115 (20), 77 (48), 57 (100). HRMS calcd for $C_{19}H_{20}N_3OSe$ [M + H]$^+$: 386.0772. Found: 386.0770.

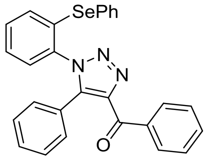

Phenyl(5-phenyl-1-(2-(phenylselanyl)phenyl)-1*H*-1,2,3-triazol-4-yl)methanone (3c). Yield: 0.123 g (85%); yellow oil. ^{1}H NMR ($CDCl_3$, 400 MHz) δ = 8.32-8.30 (m, 2H), 7.58-7.54 (m, 1H), 7.49-7.45 (m, 2H), 7.39-7.22 (m, 14H). ^{13}C NMR

[3] Savegnago, L.; Sacramento, M.; Brod, L. M. P.; Fronza, M. G.; Seus, N.; Lenardão, E. J.; Paixão, M. W.; Alves, D. *RSC Adv.* **2016**, *6*, 8021.

(CDCl$_3$, 100 MHz) δ = 186.58, 142.91, 142.27, 137.22, 135.62, 134.65, 133.31, 132.94, 132.71, 130.78, 130.61, 130.14, 129.65, 129.50, 128.70, 128.43, 128.14, 128.12, 128.10, 127.47, 125.64. MS (relative intensity) m/z: 481 (6), 376 (14), 296 (15), 152 (7), 105 (100), 77 (42). HRMS calcd for $C_{27}H_{20}N_3OSe$ [M + H]$^+$: 482.0772. Found: 482.0742.

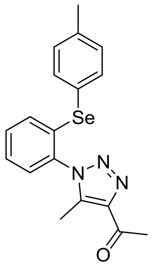

1-(5-Methyl-1-(2-(*p*-tolylselanyl)phenyl)-1*H*-1,2,3-triazol-4-yl)ethan-1-one (3f). Yield: 0.082 g (74%); White solid; mp 99-100 ºC. ^{1}H NMR (CDCl$_3$, 400 MHz) δ = 7.38-7.29 (m, 5H), 7.26-7.23 (m, 1H), 7.12 (d, *J* = 7.8 Hz, 2H), 2.76 (s, 3H), 2.45 (s, 3H), 2.35 (s, 3H). ^{13}C NMR (CDCl$_3$, 100 MHz) δ = 194.13, 143.11, 139.15, 138.49, 135.61, 134.46, 133.52, 132.46, 131.10, 130.47, 127.61, 127.41, 123.72, 27.68, 21.12, 9.63. MS (relative intensity) m/z: 371 (41), 367 (20), 300 (22), 285 (16), 252 (69), 250 (34), 232 (17), 221 (100), 208 (15), 172 (22), 165 (21), 157 (6), 152 (30), 91 (39), 77 (20), 43 (55). HRMS calcd for $C_{18}H_{18}N_3OSe$ [M + H]$^+$: 372.0615. Found: 372.0616.

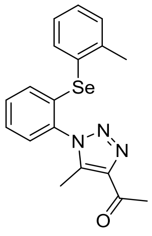

1-(5-Methyl-1-(2-(*o*-tolylselanyl)phenyl)-1*H*-1,2,3-triazol-4-yl)ethan-1-one (3g). Yield: 0.093 g (84%); yellow oil. ^{1}H NMR (CDCl$_3$, 400 MHz) δ = 7.48-7.46 (m, 1H), 7.40-7.32 (m, 2H), 7.28-7.25 (m, 3H), 7.19-7.17 (m, 1H), 7.13-7.08 (m, 1H), 2.76 (s, 3H), 2.45 (s, 3H), 2.29 (s, 3H). ^{13}C NMR (CDCl$_3$, 100 MHz) δ = 194.13, 143.12, 141.76, 138.45, 136.59, 134.69, 132.72, 132.09, 131.18, 130.60, 129.50, 128.20, 127.78, 127.40, 126.99, 27.67, 22.51, 9.56. MS (relative intensity) m/z: 371 (65), 369 (30), 300 (12), 285 (12), 272 (17), 252 (100), 232 (16), 220 (43), 204 (26), 172 (25), 165 (47), 157 (10), 144 (43), 131 (58), 91 (67). HRMS calcd for $C_{18}H_{18}N_3OSe$ [M + H]$^+$: 372.0615. Found: 372.0608.

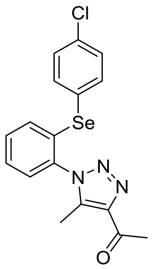

1-(1-(2-((4-Chlorophenyl)selanyl)phenyl)-5-methyl-1*H*-1,2,3-triazol-4-yl)ethan-1-one (3h). Yield: 0.102 g (87%); yellow oil. ^{1}H NMR ($CDCl_3$, 400 MHz) δ = 7.44-7.37 (m, 5H), 7.32-7.30 (m, 1H), 7.25 (d, J = 8.4 Hz, 2H), 2.75 (s, 3H), 2.45 (s, 3H). ^{13}C NMR ($CDCl_3$, 100 MHz) δ = 193.90, 142.99, 138.32, 136.11, 135.03, 134.91, 133.21, 132.01, 131.24, 129.69, 128.10, 127.69, 126.05, 27.55, 9.54. MS (relative intensity) m/z: 393 (17); 391 (39), 252 (100), 250 (52), 232 (20), 172 (17), 156 (13), 152 (43), 103 (12), 77 (20), 43 (71). HRMS calcd for $C_{17}H_{15}ClN_3OSe$ [M + H]$^+$: 392.0069. Found: 392.0047.

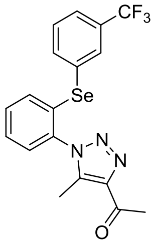

1-(5-Methyl-1-(2-((3-(trifluoromethyl)phenyl)selanyl)phenyl)-1H-1,2,3-triazol-4-yl)ethan-1-one (3i). Yield: 0.071 g (56%); yellow oil. ^{1}H NMR ($CDCl_3$, 400 MHz) δ = 7.66 (s, 1H), 7.61-7.56 (m, 2H), 7.53-7.46 (m, 5H), 7.43-7.39 (m, 1H), 7.36-7.34 (m, 1H), 2.73 (s, 3H), 2.44 (s, 3H). ^{13}C NMR ($CDCl_3$, 100 MHz) δ = 193.86, 142.98, 138.31, 137.48, 135.59, 134.18, 131.62 (q, J = 33 Hz), 131.39, 130.93, 130.52 (q, J = 3.7 Hz), 129.82, 129.51, 128.77, 127.94, 125.14 (q, J = 3.7 Hz), 123.26 (q, J = 273 Hz), 27.48, 9.51. MS (relative intensity) m/z: 425 (27), 423 (14), 275 (40), 274 (16), 254 (26), 252 (100), 250 (49), 248 (20), 232 (16), 210 (18), 152 (28), 144 (28), 77 (19), 43 (88). HRMS calcd for $C_{18}H_{15}F_3N_3OSe$ [M + H]$^+$: 426.0332. Found: 426.0309.

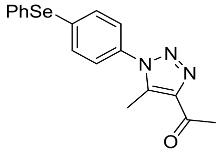

1-(5-Methyl-1-(4-(phenylselanyl)phenyl)-1*H*-1,2,3-triazol-4-yl)ethan-1-one (3j). The mixture of regioisomers was obtained in a 6:1 ratio, which was determined by ^{1}H NMR analysis. Yield: 0.099 g (92%); yellow solid; mp 74-75 ºC. ^{1}H

NMR ($CDCl_3$, 400 MHz) δ = 7.60-7.58 (m, 2H), 7.53 (d, J = 8.6 Hz, 2H), 7.36-7.30 (m, 5H), 2.71 (s, 3H), 2.56 (s, 3H). ^{13}C NMR ($CDCl_3$, 100 MHz) δ = 193.96, 143.52, 138.76, 137.12, 135.31, 134.52, 132.03, 129.59, 128.37, 126.63, 125.63, 27.60, 9.95. MS (relative intensity) m/z: 357 (10), 287 (12), 232 (16), 207 (100), 157 (10), 152 (19), 130 (11), 77 (17), 69 (15), 43 (38). HRMS calcd for $C_{17}H_{16}N_3OSe$ $[M + H]^+$: 358.0459. Found: 358.0428.

SELECTED SPECTRA

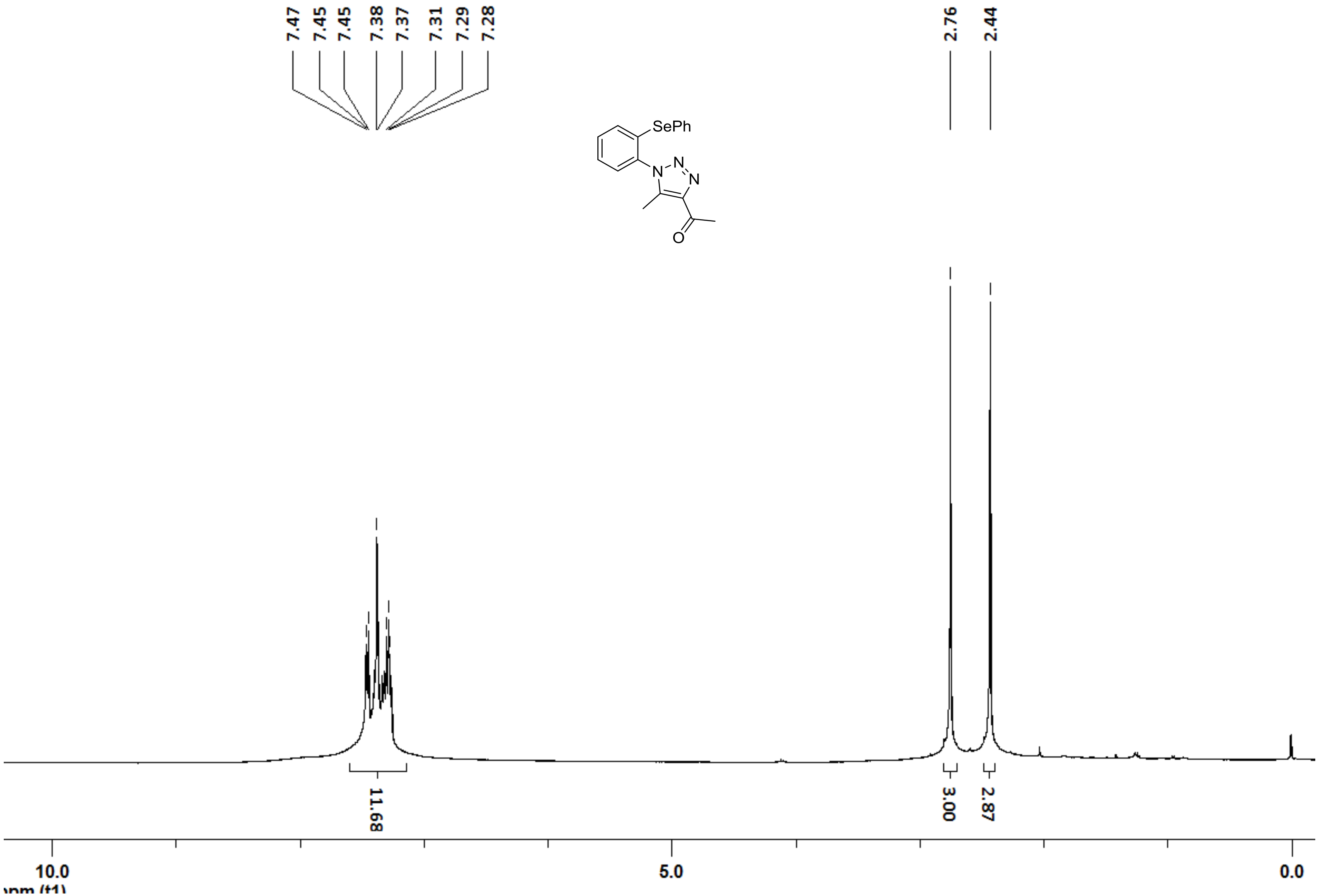

Figure 1. ^{1}H NMR (400 MHz) spectrum for compound **3a** in $CDCl_3$

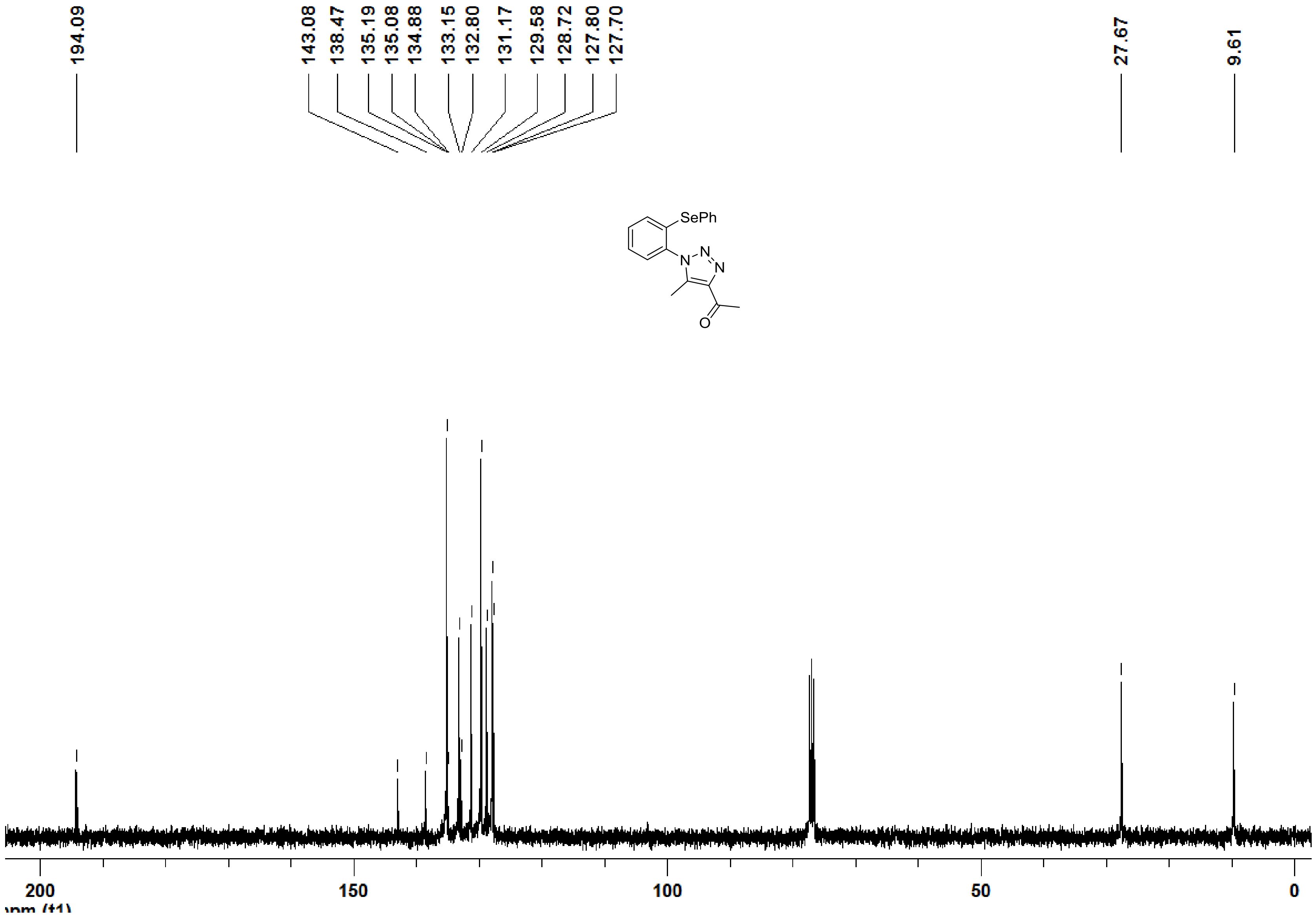

Figure 2. ^{13}C NMR (100 MHz) spectrum for compound **3a** in $CDCl_3$.

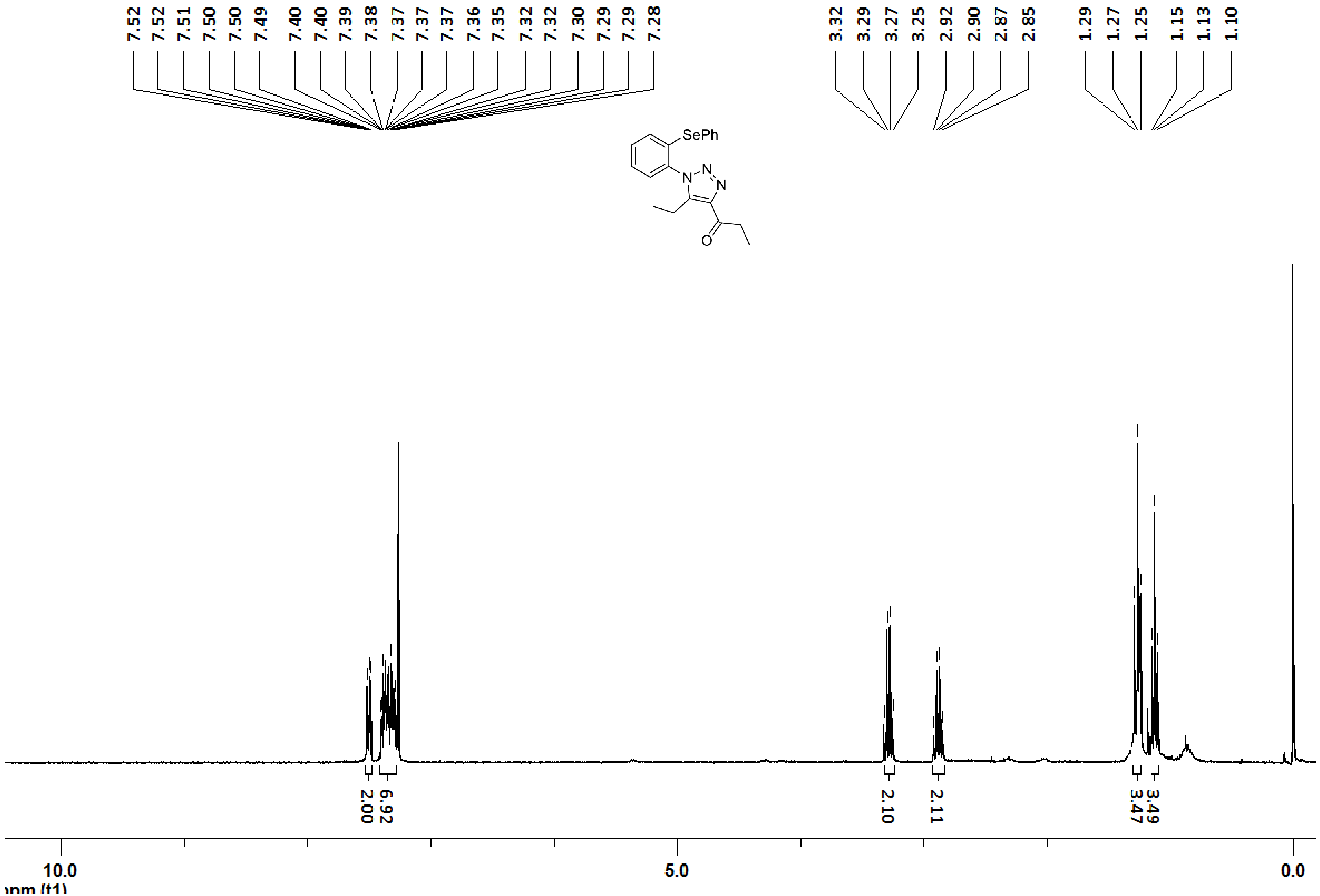

Figure 3. ^{1}H NMR (400 MHz) spectrum for compound **3b** in $CDCl_3$.

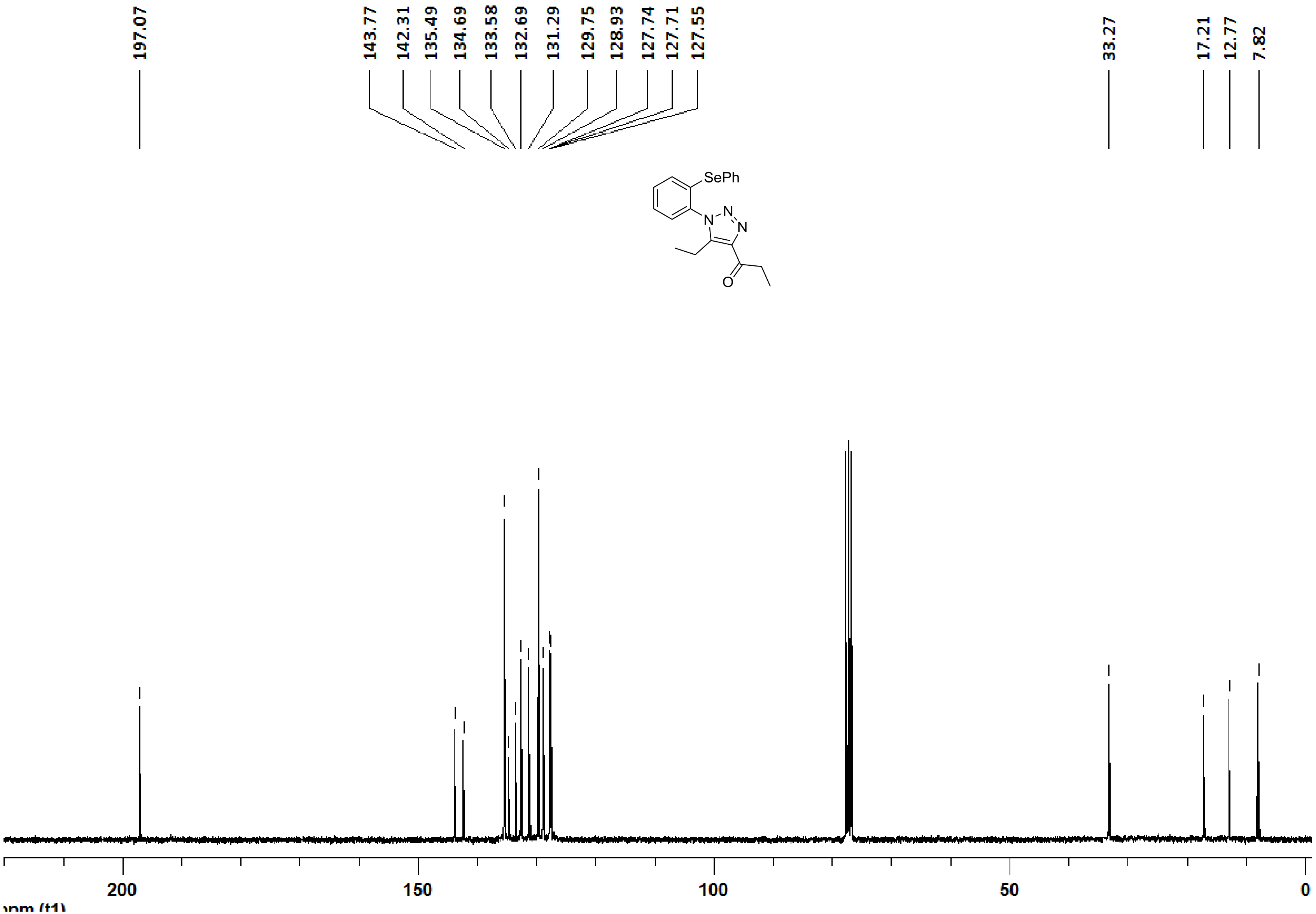

Figure 4. ^{13}C NMR (100 MHz) spectrum for compound **3b** in $CDCl_3$.

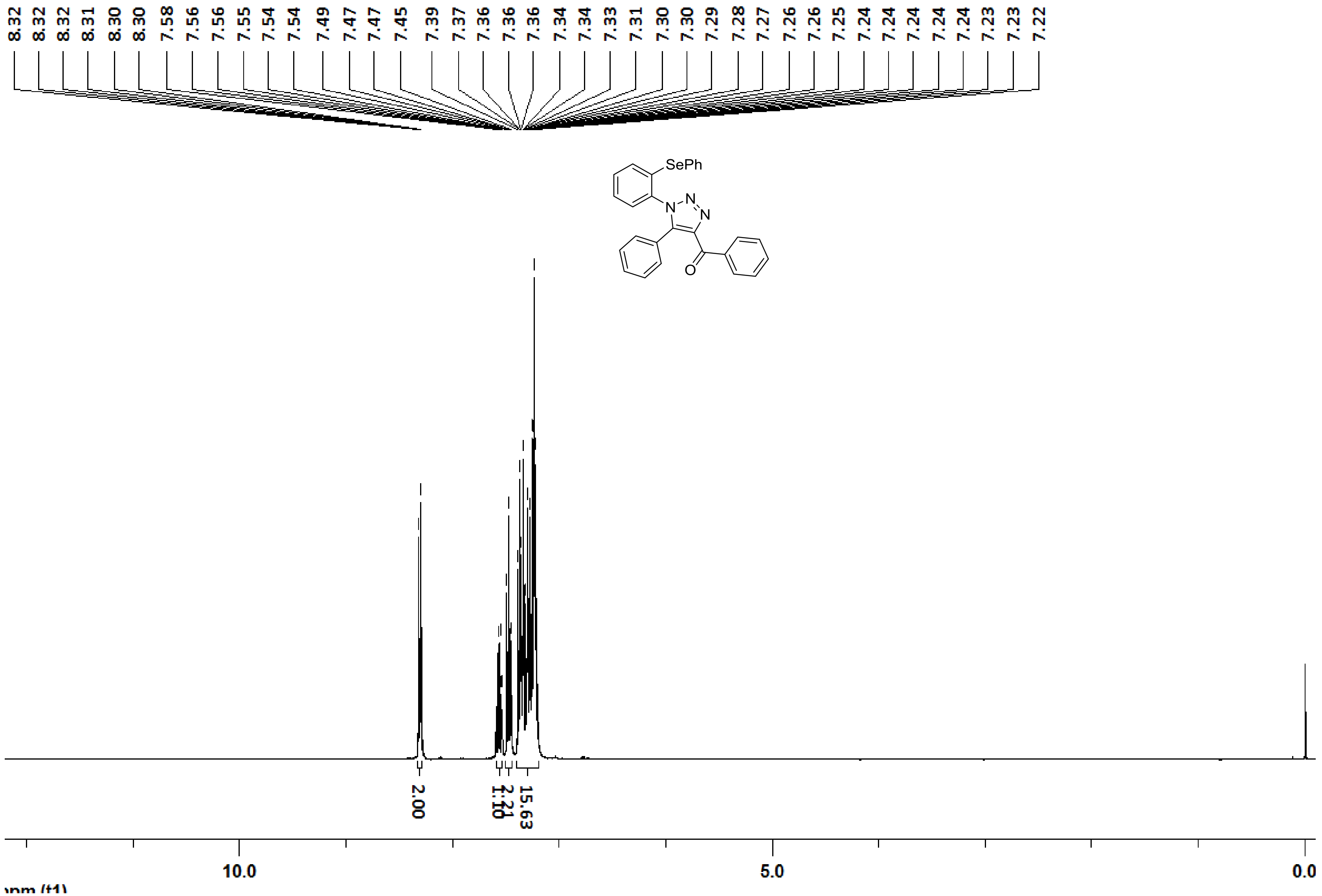

Figure 5. ^{1}H NMR (400 MHz) spectrum for compound **3c** in $CDCl_3$.

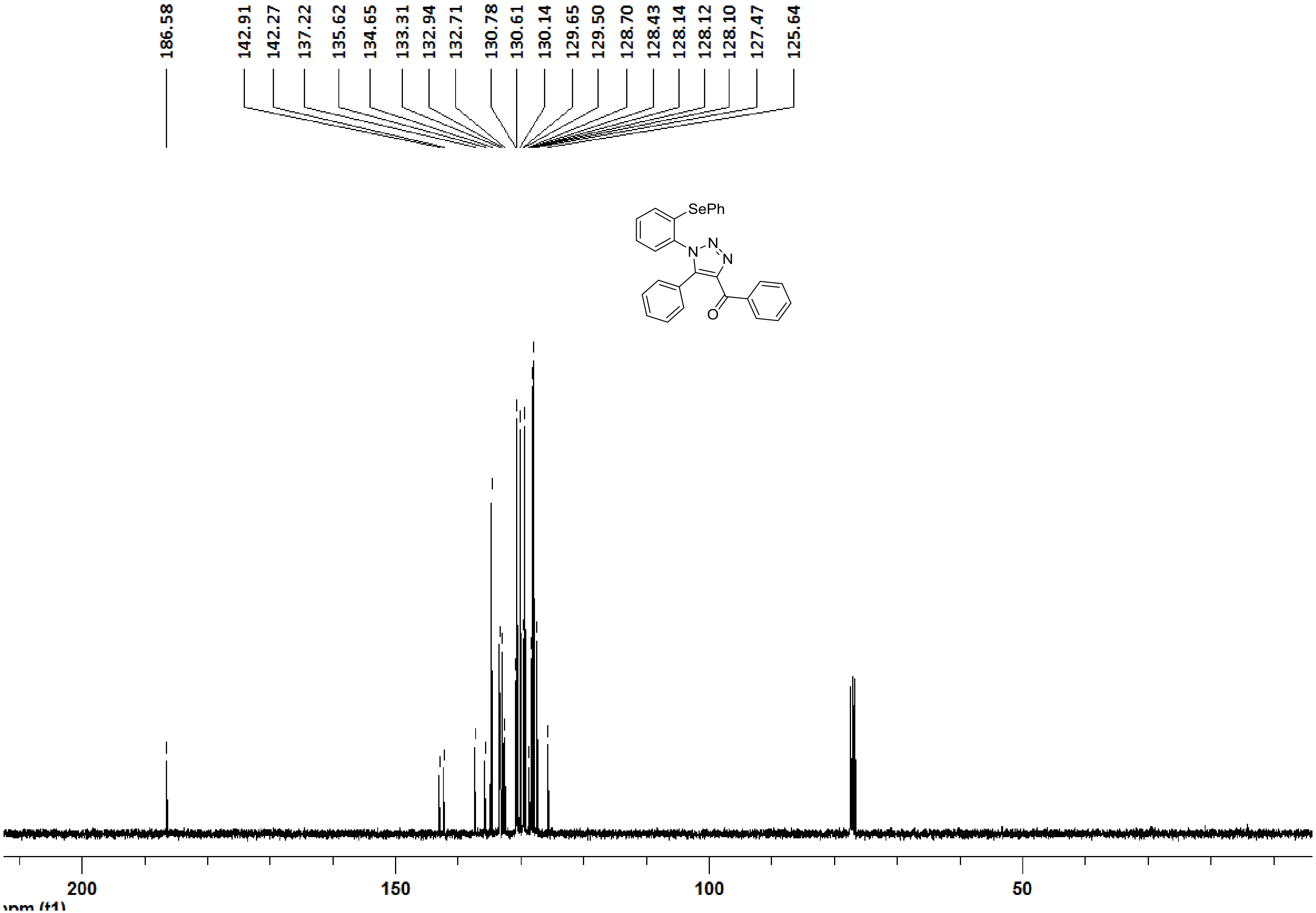

Figure 6. ^{13}C NMR (100 MHz) spectrum for compound **3c** in $CDCl_3$.

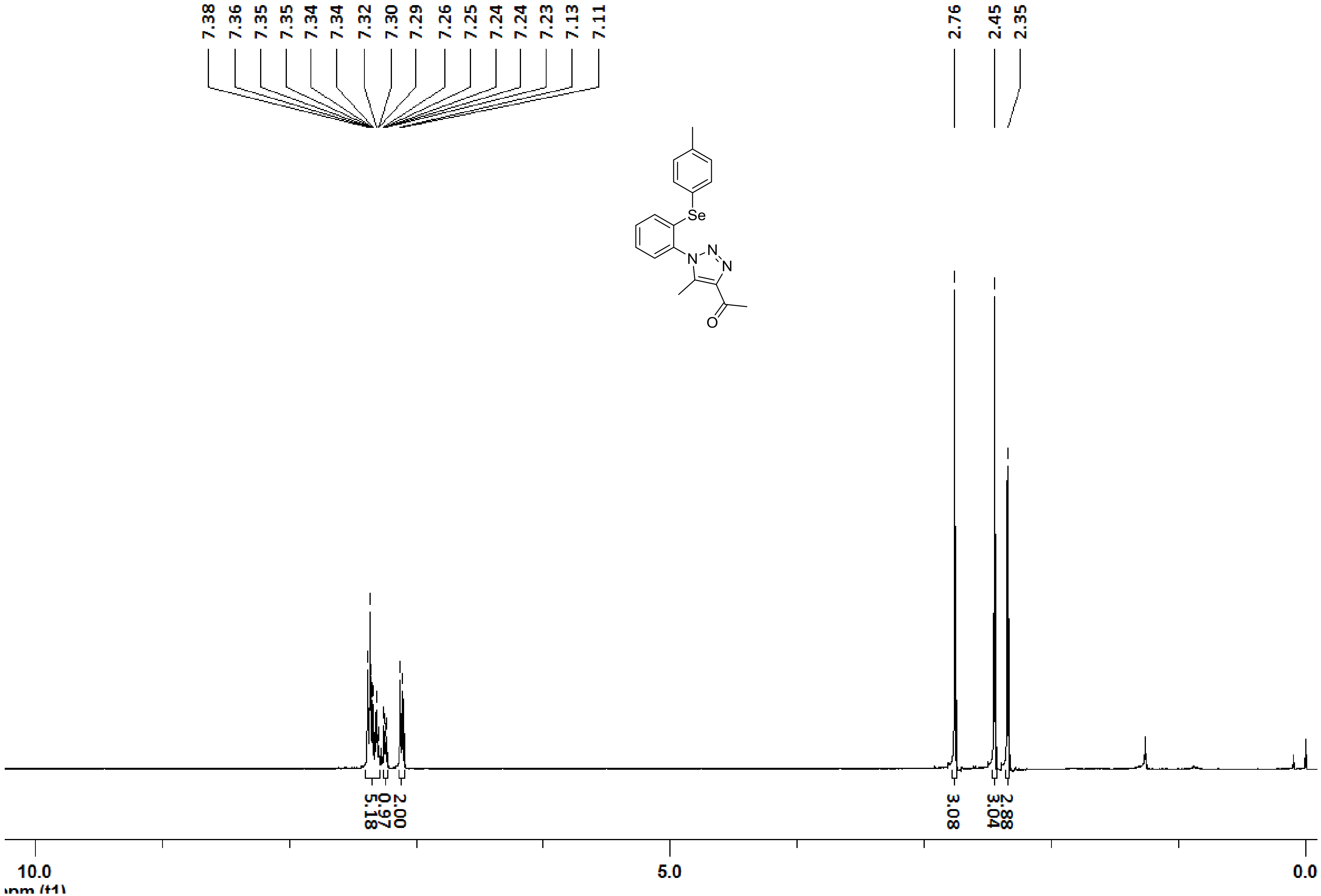

Figure 7. ^{1}H NMR (400 MHz) spectrum for compound **3f** in $CDCl_3$.

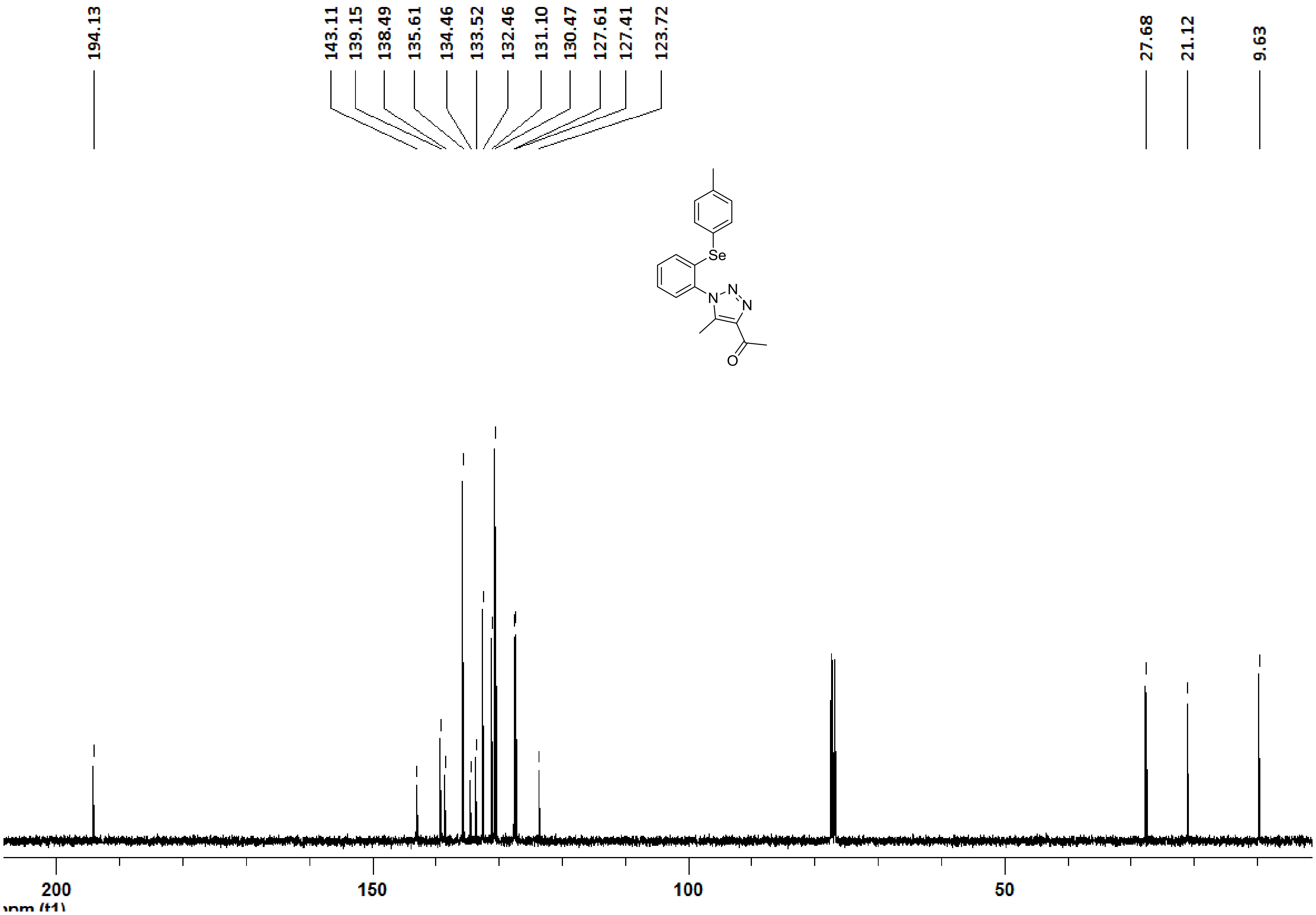

Figure 8. ^{13}C NMR (100 MHz) spectrum for compound **3f** in $CDCl_3$.

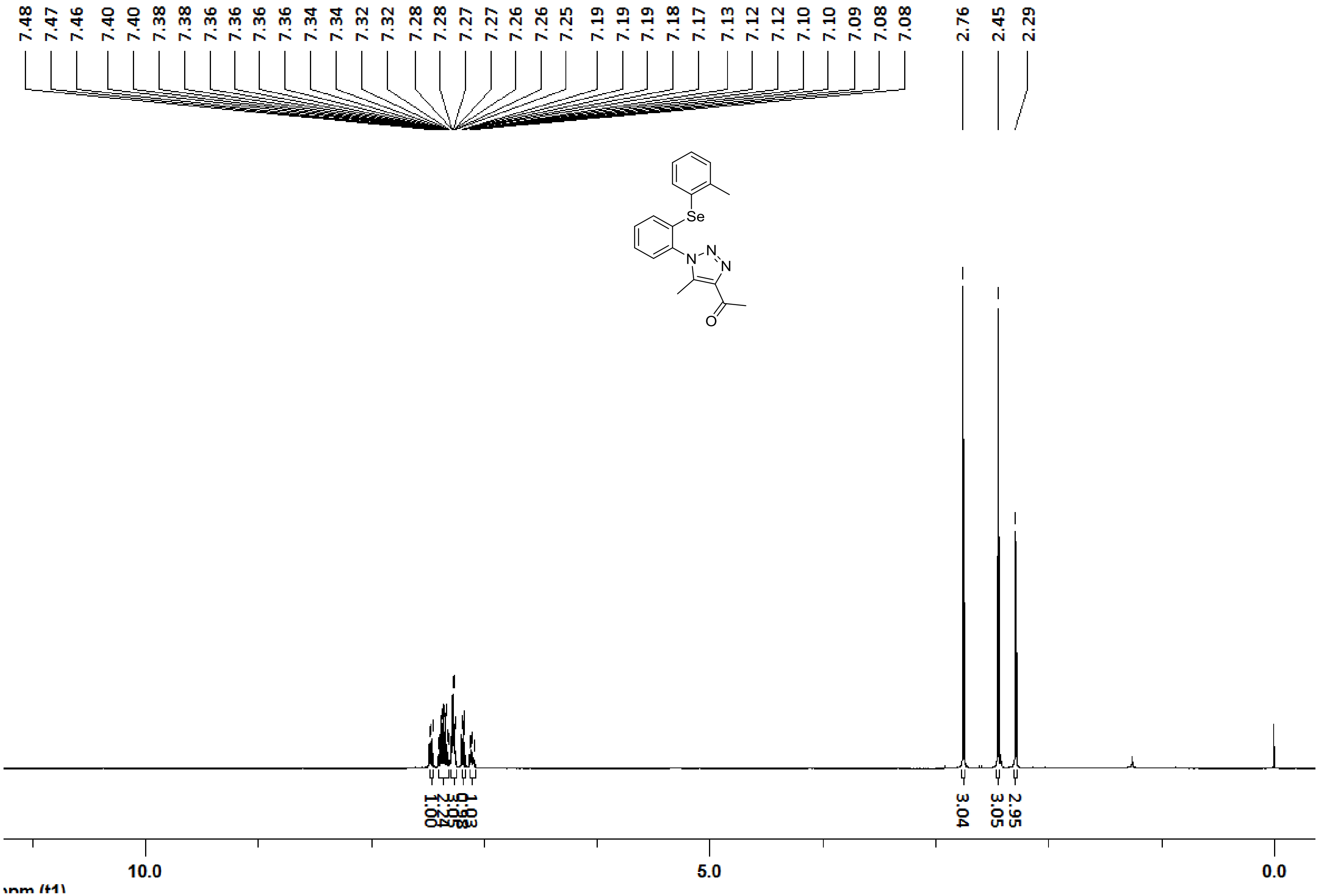

Figure 9. ^{1}H NMR (400 MHz) spectrum for compound **3g** in $CDCl_3$.

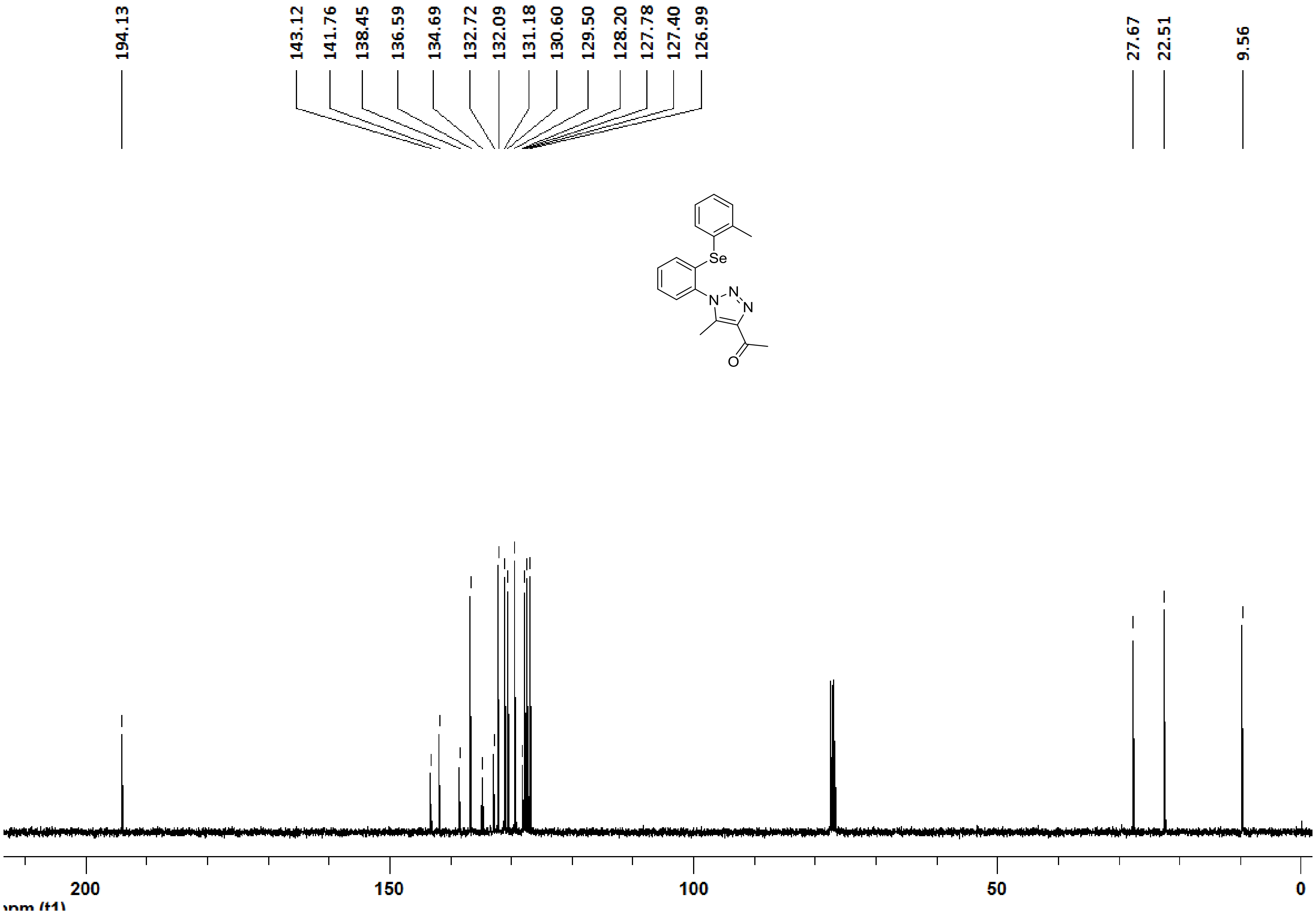

Figure 10. ^{13}C NMR (100 MHz) spectrum for compound **3g** in $CDCl_3$.

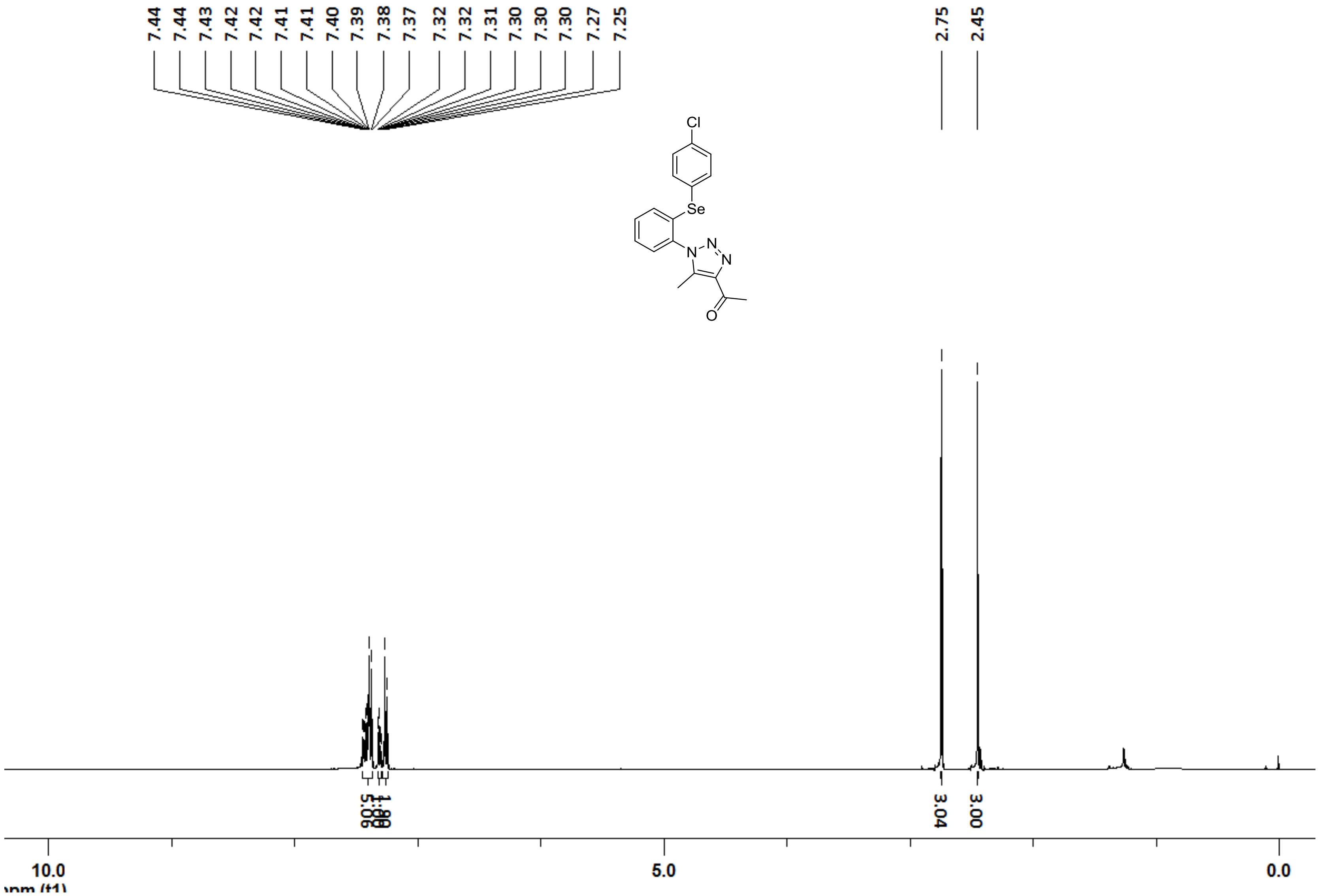

Figure 11. ^{1}H NMR (400 MHz) spectrum for compound **3h** in $CDCl_3$.

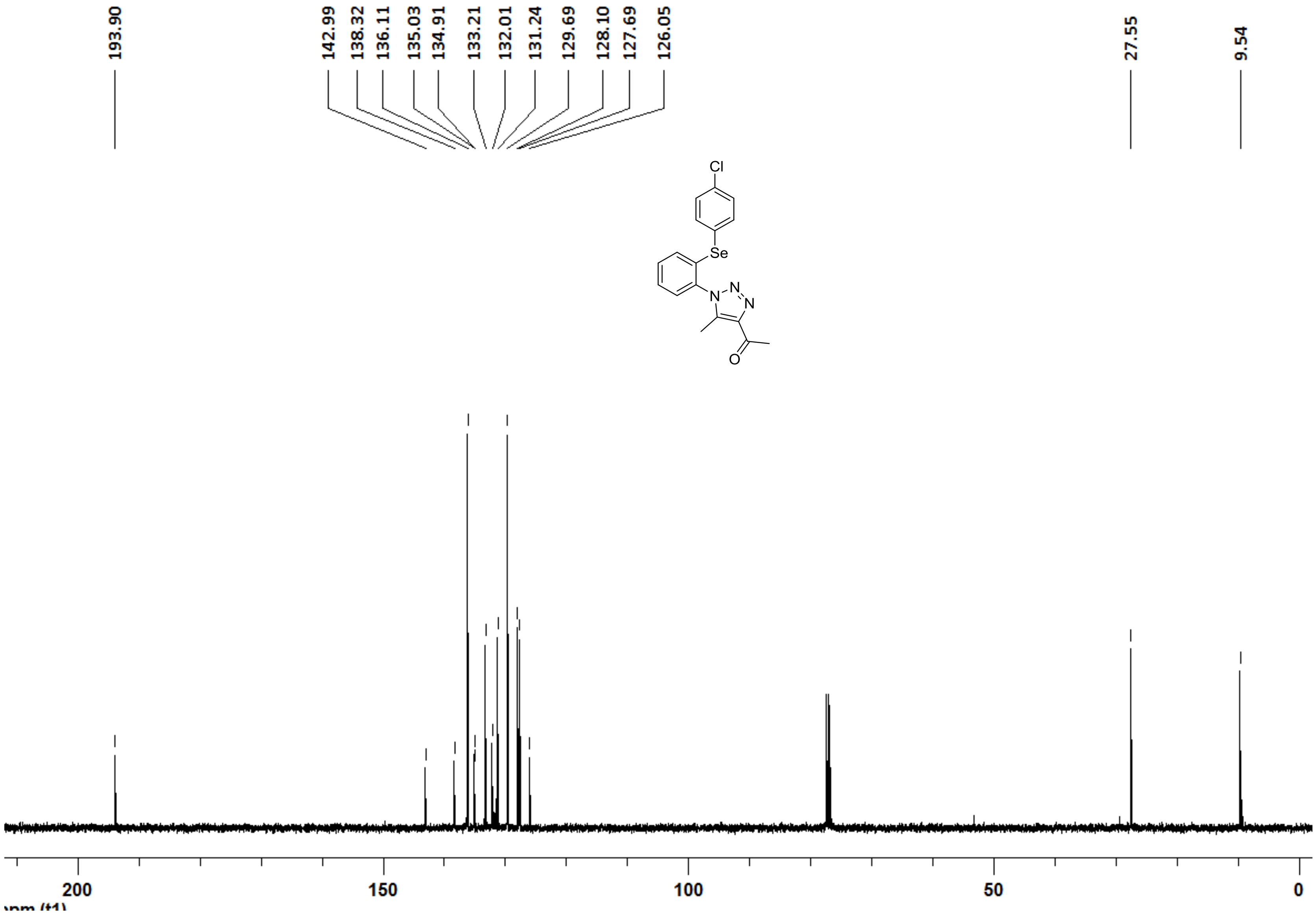

Figure 12. ^{13}C NMR (100 MHz) spectrum for compound **3h** in $CDCl_3$.

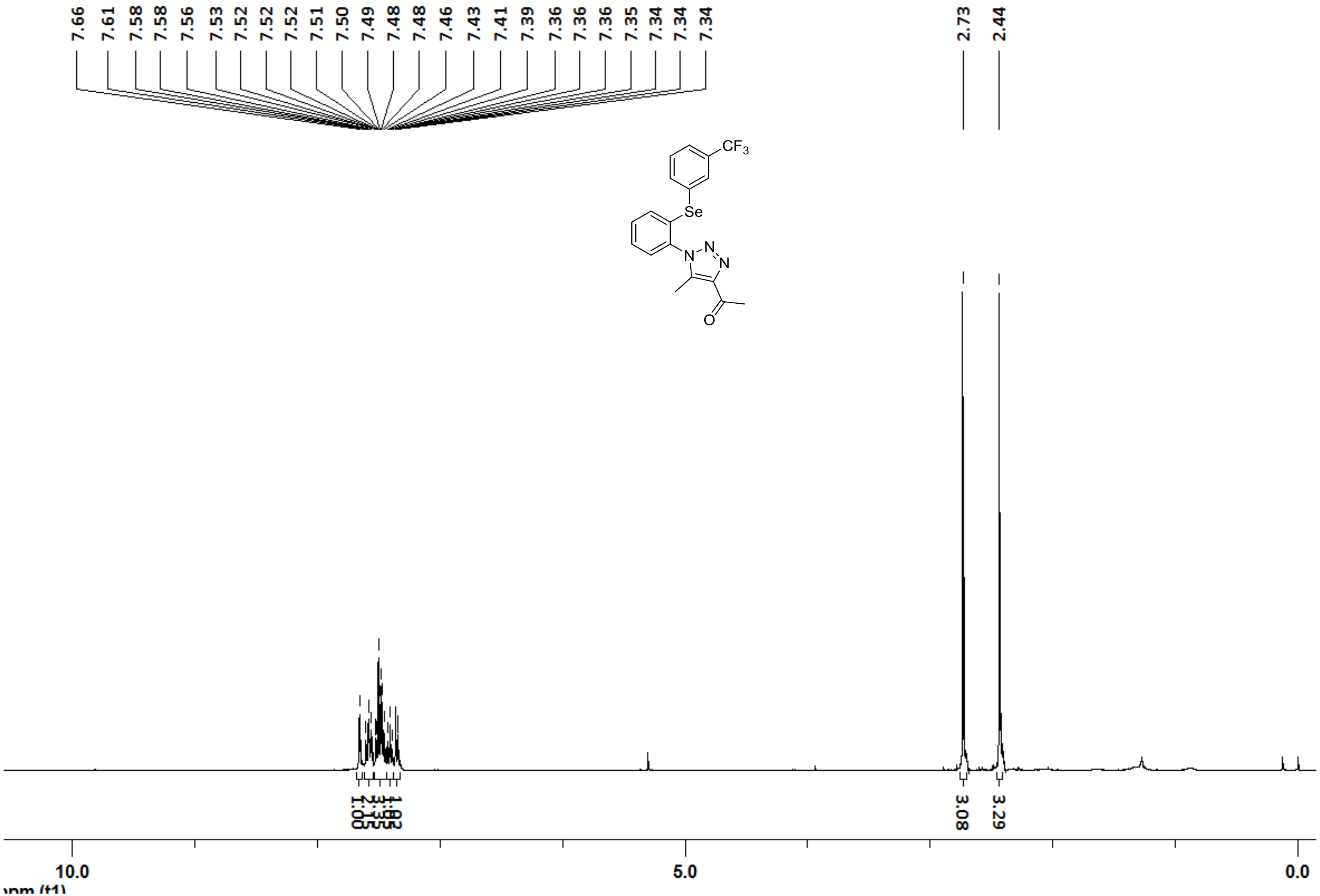

Figure 13. ^{1}H NMR (400 MHz) spectrum for compound **3i** in $CDCl_3$.

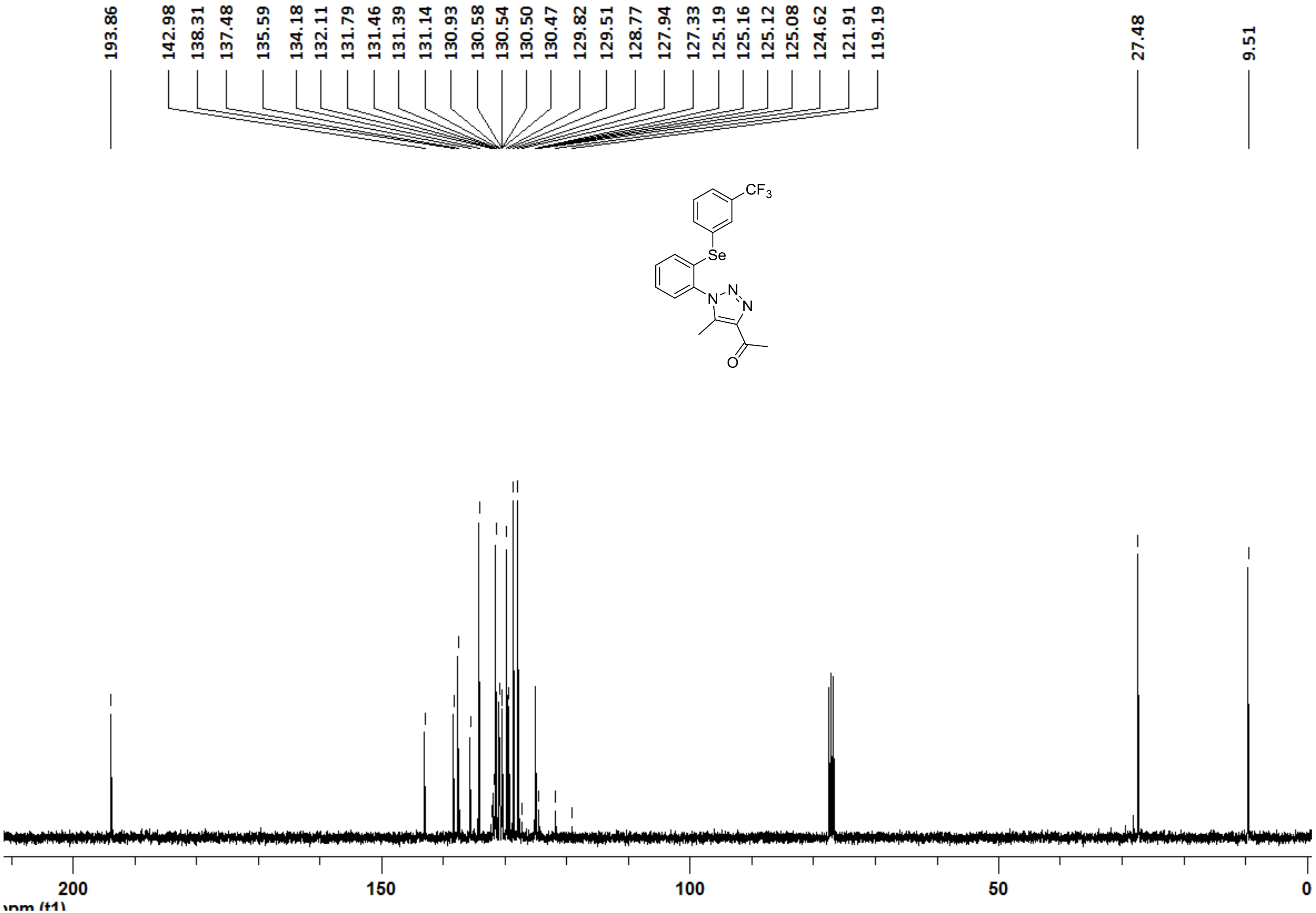

Figure 14. ^{13}C NMR (100 MHz) spectrum for compound **3i** in $CDCl_3$.

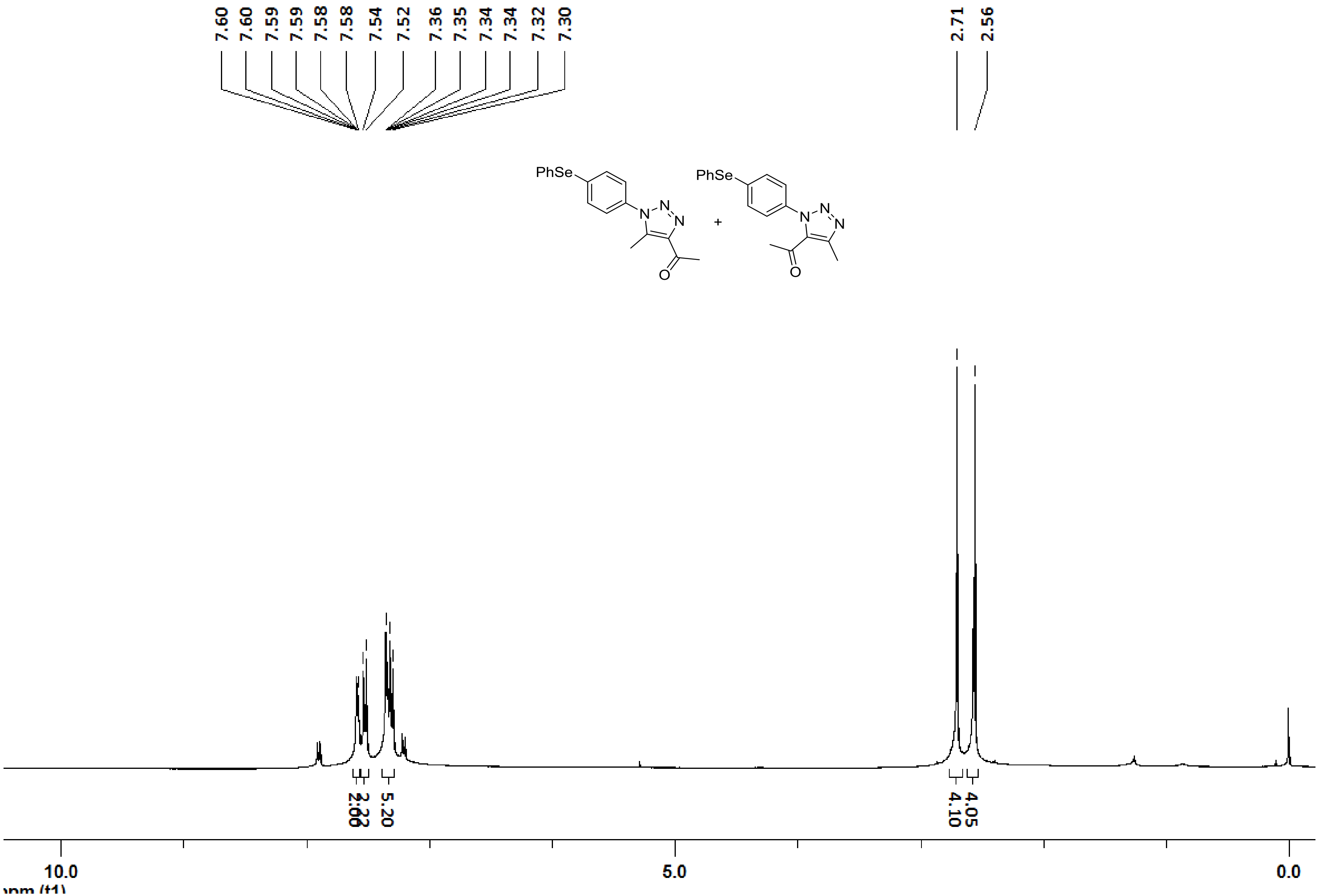

Figure 15. ^{1}H NMR (400 MHz) spectrum for compound **3j** in $CDCl_3$.

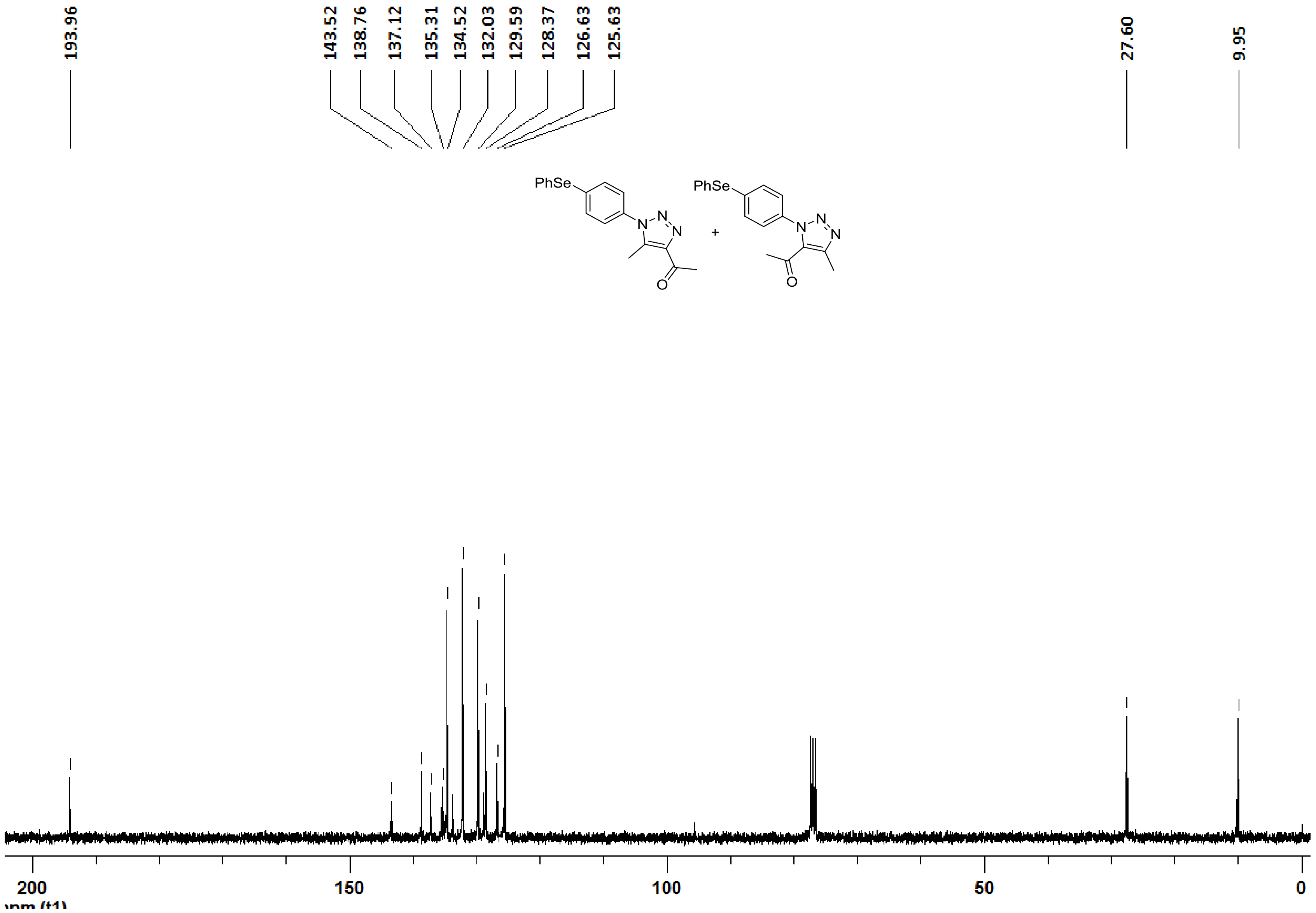

Figure 16. ^{13}C NMR (100 MHz) spectrum for compound **3j** in $CDCl_3$.
